# The Role of Marital Satisfaction and Paternal Attachment in Relation to Depression During the Transition to Fatherhood: Evidence from Turkey

**DOI:** 10.3390/healthcare14172829

**Published:** 2026-09-03

**Authors:** Emine Kurt Can, Veysel Can

**Affiliations:** Faculty of Health Sciences, Van Yüzüncü Yıl University, 65000 Van, Turkey; veyselcan@yyu.edu.tr

**Keywords:** marital satisfaction, paternal attachment, paternal depression, perinatal period, paternal involvement

## Abstract

Background: Fathers may experience psychological difficulties during the transition to parenthood, and depressive symptoms during the antenatal period may be associated with fathers’ well-being and adjustment to the paternal role. Marital satisfaction and paternal attachment may be important psychosocial factors associated with fathers’ mental health during this period. This study aimed to examine the relationships among marital satisfaction, paternal attachment, and depressive symptoms among expectant fathers. Methods: A descriptive cross-sectional study design was employed. The study was approved by the Van Yüzüncü Yıl University Non-Interventional Clinical Research Ethics Committee (20 December 2024; Approval No. 2024/13-07). Institutional permission was obtained from the hospital where the study was conducted. Written and verbal informed consent was obtained from all participants, and the study was conducted in accordance with the principles of the Declaration of Helsinki. Data were collected between January 2025 and September 2025 at a hospital located in the Eastern Anatolia Region of Türkiye. The study was completed with 336 fathers. Data collection instruments included the Personal Information Form, Marital Life Scale (MLS), Paternal–Antenatal Attachment Questionnaire (PAAQ), and Edinburgh Postpartum Depression Scale (EPDS). Multiple regression analysis and forest plot analysis were performed. Results: A statistically significant difference was observed between EPDS scores and MLS, PAAQ, educational status, spouse’s educational status, place of residence, family type, income status, spousal attendance at antenatal visits, and history of depression (*p* < 0.05). The regression model was statistically significant (F(9326) = 14.4, *p* < 0.001) and explained 26.4% of the variance in EPDS scores (R^2^ = 0.284). In the adjusted regression model, MLS, educational level, PAAQ, and family type were significantly negatively associated with EPDS scores, whereas a history of depression was significantly positively associated with EPDS scores (*p* < 0.05). Conclusions: Higher marital satisfaction and paternal attachment were associated with lower depressive symptoms among expectant fathers, whereas a history of depression was associated with higher depressive symptoms. These findings highlight the importance of considering marital and attachment-related factors when assessing fathers’ psychological well-being during the antenatal period.

## 1. Background

Father–infant bonding is one of the fundamental processes that influence a child’s cognitive, emotional, and social development in early life. Research shows that as secure bonding with the father increases, breastfeeding rates rise, cognitive delays decrease, and developmental outcomes improve [1]. Today, with socio-economic developments and changing cultural norms, fathers’ involvement in childcare has increased, which has heightened interest in fathers’ perinatal mental health and the importance of father–infant bonding [2]. Especially for first-time parents, the pregnancy and postpartum periods are a process requiring intense stress and adaptation, which can affect both the mother’s and father’s mental health and lead to changes in family dynamics [3].

Paternal bonding is a dynamic structure that begins during pregnancy and continues to develop in the postpartum period. Studies indicate that expectant fathers’ levels of attachment to the fetus are associated with active participation in the pregnancy process, prenatal experiences, and emotional preparation [4,5,6]. In particular, it is noted that prenatal interactions, such as ultrasound experiences, strengthen fathers’ emotional bond with the fetus [6]. In addition, expectant fathers’ attachment styles and mental representations of their unborn children are among the key determinants of paternal attachment [7]. Marital harmony plays a significant role in the relationship between paternal attachment and mental health. The quality of marital relationships directly influences individuals’ perception of emotional support and their parenting experiences. Studies have shown that relationship- and marriage-based interventions increase couple satisfaction and communication [4,8,9]. Similarly, high levels of harmony between partners during pregnancy positively influence both maternal and paternal bonding [3,4]. Conversely, when marital harmony is low, increased stress, conflict, and psychological issues are reported, and these negatively impact parenting processes [4,10].

Paternal depression is a mental health issue that is increasingly drawing attention during the perinatal period. The stress experienced by expectant fathers, economic responsibilities, and the process of adapting to the parenting role can contribute to the emergence of depressive symptoms [7,9]. In particular, the psychological challenges fathers face during pregnancy and the postpartum period result in consequences that affect not only the individual but also the family system. Research indicates that paternal depression may negatively impact father–infant interaction and pose risks to children’s socio-behavioral development [7].

It has been reported that psychoeducational and attachment-based interventions administered during the prenatal period improve both parents’ mental health and enhance their attachment levels [11]. Additionally, it is noted that antenatal education programs positively influence expectant fathers’ attitudes toward breastfeeding and their attachment to the fetus [12]. These findings suggest that paternal attachment is a malleable process that can be supported through appropriate interventions.

In conclusion, expectant fathers’ marital adjustment, paternal attachment, and levels of paternal depression are important variables that should be considered together in the perinatal period. However, the current literature reveals a limited number of studies that examine these three variables together. Therefore, the planned study aims to investigate the relationship between expectant fathers’ marital adjustment and paternal attachment levels and paternal depression. It is anticipated that the findings will contribute to the development of psychosocial support programs for fathers during the perinatal period and provide a significant scientific foundation for strengthening family-based care approaches.

## 2. Methods

**Study Design**: A descriptive cross-sectional study design was employed.

**Setting and Time**: The study was conducted at a hospital located in a provincial center in the Eastern Anatolia Region of Türkiye between January 2025 and September 2025. The study included expectant fathers aged 19–50 years whose partners were between 28 and 40 weeks of gestation and who had at least a primary school education. Those who were unable to speak Turkish were excluded from the study. Data were collected through face-to-face interviews, and completion of the forms took approximately 15–20 min. The questionnaires were administered in a quiet room within the hospital, ensuring a comfortable environment for participants.

### 2.1. Data Collection Methods and Instruments

Data were collected using the Personal Information Form, Marital Life Scale (MLS), Paternal-Antenatal Attachment Questionnaire (PAAQ) and Edinburgh Postpartum Depression Scale (EPDS).

### 2.2. Personal Information Form

The Personal Information Form was developed by the researchers to collect data from fathers. It includes items on the father’s age, educational level, place of residence, income level, spouse’s age, spouse’s educational level, family type, whether the pregnancy was planned, whether the spouse received treatment to conceive, the father’s involvement in prenatal follow-up visits, the sex of the baby, and a history of depression.

### 2.3. Marital Life Scale

The Marital Life Scale is a 10-item instrument developed to assess the level of satisfaction derived from the marital relationship. The scale is structured as a 5-point Likert-type measure, where responses range from 1 (lowest dissatisfaction) to 5 (highest satisfaction). The total score ranges from 10 to 50 and is calculated by summing the item scores, with higher scores indicating greater marital satisfaction [13]. For reliability assessment, the test–retest method was applied to a group with a three-month interval between the first and second administrations, yielding a reliability coefficient of 0.85. The internal consistency of the scale was found to be high, with Cronbach’s alpha coefficients of 0.88 for the male group and 0.91 for the female group [13].

### 2.4. Paternal-Antenatal Attachment Questionnaire

The Paternal-Antenatal Attachment Questionnaire, developed by Condon in 1993, was used in this study to assess fathers’ prenatal attachment levels [14]. The Turkish validity and reliability study of the scale was conducted [15]. The questionnaire consists of 16 items, each scored on a 5-point Likert scale ranging from 1 to 5. The total score ranges from 16 to 80, with higher scores indicating higher levels of paternal attachment. The Cronbach’s alpha coefficient reported by Condon was 0.80, while the Turkish adaptation demonstrated a Cronbach’s alpha of 0.79 [15].

### 2.5. Edinburgh Postpartum Depression Scale

Developed by Cox and Holden in 1987, the EPDS is used to assess the risk and severity of postpartum depression. It consists of 10 items scored on a four-point Likert scale, with a total possible score ranging from 0 to 30. A cut-off score of 12/13 indicates a risk of depression [16]. The Turkish adaptation of the EPDS was validated with a Cronbach’s alpha coefficient of 0.79 [17].

Although the EPDS was originally developed for women, its validity and reliability for assessing depressive symptoms in fathers have also been supported. A systematic review and meta-analysis demonstrated the validity of the EPDS as a screening instrument for paternal depressive symptoms [18]. In addition, a recent validity and reliability study conducted specifically in Turkish men supported its use in this population [19]. Therefore, in the present study, the EPDS was used to assess antenatal paternal depressive symptoms rather than to establish a clinical diagnosis of depression.

### 2.6. Sample Size and Power Analysis

The sample size of the study was determined using an a priori power analysis for a multiple linear regression model. The analysis was conducted using G*Power 3.1 software, selecting the “Linear multiple regression: Fixed model, R^2^ deviation from zero” option. Effect sizes for multiple regression are defined as small (f^2^ = 0.02), medium (f^2^ = 0.15), and large (f^2^ = 0.35) [20]. In this study, an effect size of f^2^ = 0.10 was used.

The number of predictors was calculated as 22, including 20 parameters obtained after dummy coding of categorical variables and two continuous variables. Based on a conservative effect size (f^2^ = 0.10), a significance level of α = 0.05, a statistical power of 1 − β = 0.90, and 22 predictors, the minimum required sample size was calculated as 336. This value also meets the commonly recommended criterion of at least 15 participants per predictor in regression analyses. A total of 382 individuals were assessed for eligibility and invited to participate in the study. Of these, 46 declined to participate, resulting in a final sample of 336 participants.

The dataset comprising 336 participants provided sufficient statistical power for conducting multiple linear regression analysis.

### 2.7. Ethical Considerations

The study was approved by the Van Yüzüncü Yıl University Non-Interventional Clinical Research Ethics Committee (20 December 2024; Approval No. 2024/13-07). Study permission was granted by the hospital where the research was conducted. Participants were informed about the study, and written and verbal consent was obtained in line with the principle of “Informed Consent.” The study adhered to the ethical principles of “Respect for Autonomy,” ensuring participants were aware of their right to participate or withdraw freely at any stage of the study, and “Confidentiality and Data Protection,” guaranteeing that all participant information would remain confidential. The research was conducted in accordance with the ethical guidelines of the Declaration of Helsinki.

### 2.8. Statistical Analysis

The study data were analyzed using IBM SPSS Statistics version 26.0. Descriptive statistics included frequency (*n*), percentage (%), mean and standard deviation ($\bar{x}$ ± SD), median, mode, and minimum–maximum values. Relationships between variables were examined using Pearson correlation analysis; correlation coefficients were interpreted as low (0.10–0.29), moderate (0.30–0.49), and high (≥0.50). The internal consistency of the scales was assessed using Cronbach’s alpha coefficient, with values ≥0.70 considered acceptable. A 95% confidence interval was adopted in all analyses, and *p* < 0.05 was considered statistically significant. Effect sizes for significant differences were calculated using eta-squared (η^2^) and interpreted as small (0.01), medium (0.06), and large (0.14). The assumption of normality was evaluated based on skewness and kurtosis values; values within the range of ±2.0 were considered indicative of normal distribution. When parametric test assumptions were met, an independent sample *t*-test was used for comparisons between two groups, and one-way analysis of variance (ANOVA) was used for comparisons among three or more groups. In cases where assumptions were not met, the Kruskal–Wallis H test was applied. Pairwise comparisons between groups were conducted using Bonferroni-adjusted post hoc analysis. To ensure comparability of scale scores with different item numbers and scoring ranges, raw scores were standardized using the Percentage of Maximum Possible (POMP) method and transformed to a 0–100 scale [21]. Multiple linear regression analysis with the enter method was performed to identify variables predicting Edinburgh Postpartum Depression Scale (EPDS) scores. The significance of regression coefficients was assessed using *t*-tests, and standardized beta coefficients (β) were reported to indicate relative effects. To construct standard confidence intervals in the forest plot, standard errors of standardized beta coefficients were calculated using the delta method, and 95% confidence intervals were generated. A forest plot was used to visually present regression beta coefficients and their confidence intervals.

## 3. Results

The sociodemographic and obstetric characteristics of the participants are presented in Table 1.

Descriptive statistics of the scales are presented in Table 2.

As shown in Table 3, a statistically significant difference was found between MLS scores and age, educational status, spouse’s educational status, place of residence, family type, income status, planned pregnancy status, complications in the spouse’s pregnancy, spouse’s attendance at antenatal visits, and history of depression (*p* < 0.05).

A statistically significant difference was found between PAAQ scores and age, spouse’s age, educational status, spouse’s educational status, place of residence, family type, income status, planned pregnancy status, complications during pregnancy, and spouse’s attendance at antenatal visits (*p* < 0.05).

A statistically significant difference was found between EPDS scores and educational status, spouse’s educational status, place of residence, family type, income status, spouse’s attendance at antenatal visits, and history of depression (*p* < 0.05).

As shown in Table 4, the model constructed using the Enter method was statistically significant overall (F(9, 326) = 14.4, *p* < 0.001). The nine independent variables collectively explained 26.4% of the variance in EPDS scores (Adjusted R^2^ = 0.264). The evaluation of model fit demonstrated favorable information criteria values (AIC= 2000, BIC= 2042). Additionally, residual independence was confirmed by the Durbin–Watson statistic (2.09, *p* = 0.386) and multicollinearity was not a concern, as Variance Inflation Factor (VIF) values ranged between 1.06 and 3.14 (well below the conservative threshold of 5.0), and Tolerance values ranged between 0.318 and 0.945 (well above the 0.10 threshold).

Although the model was statistically significant overall, the regression coefficients of the individual independent variables were also examined.

MLS showed the strongest negative association with EPDS scores, followed by educational level and PAAQ (*p* < 0.05). Family type was also significantly negatively associated with EPDS scores (*p* < 0.05), whereas a history of depression was significantly positively associated with EPDS scores (*p* < 0.05).

**Figure 1:**
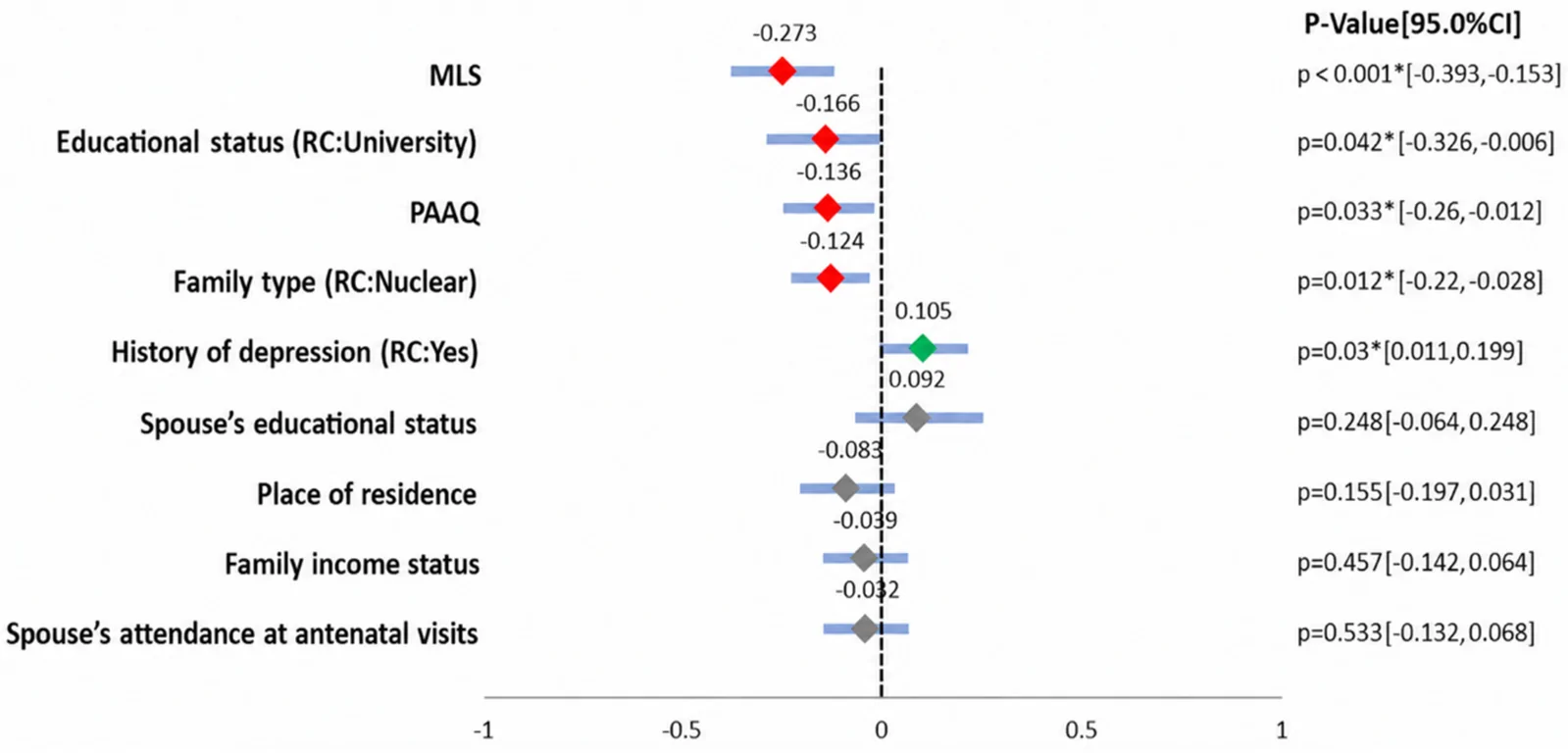
Forest plot of predictors of EPDS. 95% CI: 95% confidence interval, * *p* < 0.05, MLS: Marital Life Scale, PAAQ: Paternal-Antenatal Attachment Questionnaire.

As shown in Figure 1, diamonds represent standardized beta coefficients (β), and the horizontal light blue lines indicate the 95% confidence intervals (95% CI) for the standardized beta coefficients. RC denotes the reference category. The vertical dashed line represents the zero reference line; red diamonds indicate negative coefficients, green diamonds indicate positive coefficients, and gray diamonds indicate statistically non-significant coefficients. A forest plot was used to visually present the variables associated with EPDS scores. The most prominent feature of the graph is that the majority of variables are clustered on the left side of the zero reference line (negative region), indicating negative associations with EPDS scores. MLS appears at the far left of the graph, showing the strongest negative association with EPDS scores. Other variables, such as educational level, PAAQ, and family type, are also represented by red diamonds; however, their associations are weaker than that of MLS, as their confidence intervals are closer to the zero line. Higher educational attainment (university level) and living in a nuclear family structure were associated with lower EPDS scores. The variable history of depression, represented by the only green diamond, is located on the right side of the zero line, indicating a positive association with antenatal paternal depressive symptoms. Gray diamonds denote predictors whose confidence intervals include zero, indicating that their regression coefficients were not statistically significant. Accordingly, variables such as spouse’s educational status, place of residence, family income status, and spouse’s attendance at antenatal visits were not significantly associated with EPDS scores in the adjusted model. Although some of these variables have negative beta coefficients (e.g., place of residence: −0.083), their confidence intervals include zero. In conclusion, the forest plot visualization shows that MLS, educational level, PAAQ, family type, and history of depression were significantly associated with EPDS scores in the adjusted model.

## 4. Discussion

In this study, marital satisfaction, paternal attachment, and depression levels of fathers during the perinatal period were examined within a holistic framework in relation to individual, sociodemographic, and psychosocial variables. The findings indicate that these three core constructs are closely associated with one another and that paternal mental health is associated not only with individual characteristics but also with family and environmental factors. In this context, the results were evaluated in light of the existing literature, and similarities and differences were discussed.

Marital satisfaction is a dynamic construct related to individual, relational, and environmental factors. The pregnancy period, in particular, is a significant process during which couples’ roles and relationship dynamics are reorganized. During this period, the associations between various sociodemographic and psychosocial factors and marital harmony may become more pronounced [4,22,23].

In this study, fathers’ age, educational status, spouse’s educational level, place of residence, family type, and income level were found to be associated with marital satisfaction, highlighting the relevance of individual, sociodemographic, and environmental factors. In particular, fathers’ age, education, and income level were associated with communication and living conditions, whereas spouses’ education, family type, and place of residence were associated with relationship dynamics. These findings are consistent with previous research and support the multidimensional nature of marital satisfaction [4,24,25]. In our study, fathers with a history of depression had lower marital harmony. This finding is supported by the literature [23].

Marital satisfaction was higher among fathers with higher educational levels and those whose spouses had higher educational levels. Previous studies support our finding that a higher level of education is associated with more positive parent–child relationships [4,20,24]. The observed association may reflect differences in socioeconomic resources, communication skills, or relationship dynamics.

It was observed that marital satisfaction was higher among fathers with higher income, a finding consistent with the literature [22,26]. Similarly, it has been reported that fathers living in urban areas had higher marital satisfaction scores compared to rural residents [20]. Furthermore, the finding that marital harmony was higher among individuals in nuclear families aligns with existing studies [27]. These findings suggest that economic conditions and characteristics of the living environment may be associated with relationship dynamics.

In our study, it was observed that fathers whose partners experienced a planned pregnancy had higher marital satisfaction. This finding is consistent with studies reporting that planned pregnancy is associated with higher parental harmony among fathers [4,23]. This association may reflect greater preparedness and mutual agreement between couples during the transition to parenthood.

In the present study, fathers whose spouses experienced pregnancy complications were found to have lower marital adjustment compared with those whose spouses did not. This finding is consistent with studies reporting an association between health problems during pregnancy and greater psychological burden and stress among couples [28]. However, some studies have reported higher levels of supportive coping strategies among partners experiencing high-risk pregnancies, which may be associated with marital adjustment [29]. These findings suggest that the association between pregnancy complications and marital adjustment may differ according to couples’ coping mechanisms.

Our finding that fathers who accompanied their partners to prenatal checkups had higher marital harmony is consistent with the literature [30]. This association may reflect differences in mutual support and involvement in the pregnancy process.

Paternal bonding is a dynamic construct that develops during pregnancy and is related to individual, relational, and environmental factors. Paternal attachment differed according to several sociodemographic characteristics during this process [31,32]. The study found that attachment scores were higher among older fathers and among fathers whose partners were aged 25–34 years. These findings are consistent with the literature [3,33]. Furthermore, fathers with higher educational attainment and higher family income had higher attachment scores, and these findings are also supported by previous studies [31]. This suggests that individual and socioeconomic characteristics may be associated with differences in paternal attachment.

In the current literature, the association between place of residence (urban/rural) and father–fetus bonding during the prenatal period has not been demonstrated consistently. A study conducted in Türkiye found no significant association [34]. However, paternal bonding has been reported to be associated with factors such as family functioning, participation in the prenatal process, and sociodemographic characteristics [32]. These findings provide context for our observation that fathers living in urban areas reported higher attachment scores.

In our study, fathers living in nuclear families had higher levels of fetal attachment compared with those living in extended families, a finding consistent with the literature [32]. Additionally, higher attachment levels among fathers with planned pregnancies and those who accompanied their partners to prenatal checkups are consistent with previous studies [35]. These findings suggest that nuclear family structure may be associated with fathers’ involvement in the parenting process and their interactions with their partners. Similarly, planned pregnancy and fathers’ involvement in prenatal care may be associated with a more shared experience of pregnancy and greater emotional closeness. In this context, family structure and active participation in the prenatal process may be associated with father–fetus bonding. Paternal attachment may also be influenced by the sociocultural context in which fatherhood is experienced. In family-oriented cultural contexts such as Türkiye, expectations regarding paternal roles, family relationships, and fathers’ involvement during pregnancy may differ from those in more individualistic Western societies. Such cultural differences may shape how fathers perceive and express attachment and how they participate in the pregnancy process. Therefore, the relationships observed among paternal attachment, marital satisfaction, and depressive symptoms should be interpreted within the sociocultural context in which paternal roles and family relationships are experienced.

In our study, fathers whose partners experienced a high-risk pregnancy were found to have lower levels of prenatal attachment compared with those whose partners had uncomplicated pregnancies. This finding suggests that high-risk pregnancy may be negatively associated with prenatal attachment. The literature also supports this finding [36].

In the present study, paternal depressive symptoms were significantly associated with sociodemographic factors such as educational status, the spouse’s educational level, place of residence, family type, and income level. These univariate findings suggest that paternal depressive symptoms vary according to several sociodemographic characteristics. In particular, lower educational and income levels and disadvantaged living conditions may be associated with higher depressive symptoms and greater levels of stress and feelings of inadequacy among fathers. Similarly, environmental factors such as extended family structure or rural residence may be associated with greater psychosocial burden related to role expectations and social pressures. Indeed, studies conducted both in Türkiye and internationally have reported associations between socioeconomic and demographic variables and paternal depressive symptoms [11,37], consistent with the findings of the present study. These findings highlight the importance of considering family and social contexts, in addition to individual characteristics, when assessing fathers’ mental health.

Our study found that fathers who participated in prenatal checkups had significantly lower EPDS scores. This association may reflect greater involvement in the pregnancy process. Previous research has similarly reported associations between fathers’ participation during the pregnancy and postpartum periods, paternal mental health, and father–infant bonding [38].

When all candidate variables were entered simultaneously into the multiple regression model, marital satisfaction, paternal attachment, educational status, family type, and history of depression remained independently associated with EPDS scores. In our study, a history of depression was significantly associated with higher paternal depressive symptoms. This finding is consistent with previous studies reporting an association between a history of mental health problems and depressive symptoms during the transition to parenthood [11,37,39,40]. These findings suggest that previous psychiatric history should be considered alongside current symptoms when assessing fathers’ mental health during the antenatal period.

The current study found that lower marital satisfaction was significantly associated with higher paternal depressive symptoms. This finding indicates a close relationship between fathers’ mental health and the quality of their marital relationships. Lower educational and income levels may also be associated with higher depressive symptoms and greater psychological burden related to communication difficulties, limited emotional support, and interpersonal conflict. Indeed, a study conducted in Türkiye reported a significant association between marital satisfaction and paternal depressive symptoms [41]. Similarly, studies conducted in Pakistan [39] and Iran [28] reported associations between lower marital harmony and paternal depressive symptoms. Studies conducted in China have also reported similar findings [42]. Overall, these findings highlight the importance of considering the quality of the marital relationship when assessing fathers’ mental health during the antenatal period.

Our study found that higher paternal attachment was significantly associated with lower EPDS scores. This finding suggests a relationship between paternal attachment and fathers’ psychological well-being during the antenatal period. Previous studies conducted in Sweden [43], Germany [44], and France [38] have similarly reported associations between paternal attachment and depressive symptoms. Taken together, these findings highlight the importance of considering paternal attachment when assessing fathers’ psychological well-being during the antenatal period.

This study has several limitations. First, as the research was conducted using a cross-sectional design, relationships between variables could be identified; however, causal inferences cannot be established. In addition, the data were based on self-reports, which may introduce recall bias and social desirability bias, particularly in sensitive domains such as mental health and relationship satisfaction. Second, the study was conducted in a single center with fathers who attended a university hospital, which may have resulted in a sample limited to individuals with access to healthcare services and specific sociodemographic characteristics. Furthermore, the inclusion of only fathers who were Turkish-speaking, at least primary school graduates, and within a defined age range may have limited the representation of individuals with lower educational levels or restricted access to healthcare. Accordingly, the findings should be interpreted within the context of fathers who attended the healthcare institution where the study was conducted and who share similar characteristics; their generalizability to populations with different sociodemographic profiles, healthcare access levels, or cultural contexts is limited. Nevertheless, the use of validated and reliable measurement tools, along with an adequate sample size, strengthens the internal validity of the study. However, further research employing multi-center and longitudinal designs across diverse regions is needed to enhance the generalizability of the findings and to provide more robust evidence regarding causal relationships.

## 5. Conclusions

The findings of this study indicate that psychosocial processes related to fathers in the perinatal period do not operate independently but are related to multiple interconnected factors. A multidimensional and intertwined relationship was observed among marital satisfaction, paternal attachment, and paternal depressive symptoms, with higher marital satisfaction and paternal attachment being associated with lower depressive symptoms. Sociodemographic characteristics such as higher education and income levels, as well as active involvement in the pregnancy process, including planned pregnancy and participation in prenatal follow-ups, were associated with marital satisfaction and paternal attachment. In contrast, educational status, spouse’s educational status, place of residence, family type, income status, spouse’s attendance at antenatal visits, and history of depression were associated with paternal depressive symptoms. Notably, the inverse relationship between paternal attachment and depressive symptoms, as well as the association between paternal involvement in the pregnancy process and fathers’ psychological well-being, highlight potentially important areas for consideration in perinatal care. These findings suggest that fathers in the perinatal period should not be viewed merely as supportive figures but also as a population whose psychosocial needs should be considered in perinatal care. Accordingly, these findings highlight the importance of considering fathers’ psychosocial needs, including marital relationships, paternal attachment, and prior psychiatric history, within prenatal care. In conclusion, approaches to paternal mental health should extend beyond individual-level considerations and adopt a holistic perspective that includes marital relationships, parenting experiences, and engagement in the pregnancy process. Such an integrated approach may provide a broader framework for addressing paternal attachment and fathers’ psychosocial needs within family-centered perinatal care.

## Figures and Tables

**Table 1:** Participant characteristics (n = 336).

Variables	Categories	n	%
Age	19–24	18	5.4
	25–34	274	81.5
	35 +	44	13.1
Spouse’s age	19–24	106	31.5
	25–34	224	66.7
	35 +	6	1.8
Educational status	Primary education	46	13.7
	High school	150	44.6
	University	140	41.7
Spouse’s educational status	Primary education	84	25
	High school	128	38.1
	University	124	36.9
Place of residence	Urban	190	56.5
	Rural	146	43.5
Family type	Nuclear	268	79.8
	Extended	68	20.2
Employment status	Working	296	88.1
	Not working	40	11.9
Family income status	Income is less than expenses	96	28.6
	Income equals expenses	164	48.8
	Income is more than expenses	76	22.6
Duration of marriage	1–5 years	296	88.1
	5–10 years	28	8.3
	10 +	12	3.6
Planned pregnancy status	Yes	274	81.5
	No	62	18.5
Complications in the spouse’s pregnancy	Yes	46	13.7
	No	290	86.3
Infant’s sex	Female	172	51.2
	Male	164	48.8
Spouse’s attendance at antenatal visits	Yes	290	86.3
	No	46	13.7
History of depression	Yes	38	11.3
	No	298	88.7

n: number/frequency; %: percentage.

**Table 2:** Descriptive findings and correlations of the scales.

	α	$\bar{x}$ ± SD	POMP	Min-Max	Mod-Medyan	MLS	PAAQ
EPDS	0.844	8.4 ± 5.4	%28	0–23	5–8	r: −0.452	r: −0.399
						*p* < 0.001 *	*p* < 0.001 *
						η^2^: 0.204	η^2^: 0.159
MLS	0.882	37.9 ± 6.7	%69.8	19–50	40–38		r: 0.612
							*p* < 0.001 *
							η^2^: 0.375
PAAQ	0.850	59.4 ± 9.6	%67.8	41–79	71–59		

$\bar{x}$ ± SD: mean ± standard deviation, POMP: Percentage of Maximum Possible, calculated based on min-max values, α: Cronbach Alpha, r: Pearson’s correlation, η^2^: Eta-squared effect size, * *p* < 0.001, MLS: Marital Life Scale, PAAQ: Paternal-Antenatal Attachment Questionnaire, EPDS: Edinburgh Postpartum Depression Scale.

**Table 3:** Comparison of MLS, PAAQ, and EPDS scores according to variables.

	MLS			PAAQ			EPDS		
	$\bar{x}$ ± SD	Statistics	*p*/η^2^	$\bar{x}$ ± SD	Statistics	*p*/η^2^	$\bar{x}$ ± SD	Statistics	*p*/η^2^
**Age**									
19–24 (1)	34.3 ± 6	KW: 7.07	0.029 *	51.8 ± 9.9	F = 6.37	0.002 *	8.4 ± 5.7	F = 0.004	0.996
25–34 (2)	38.1 ± 6.6	^2 > 1^ **	0.02	59.8 ± 9.7	^2 > 1^ **	0.04	8.4 ± 5.4		
35+ (2)	38.5 ± 7.7			60.3 ± 7.4			8.3 ± 5.7		
**Spouse’s age**									
19–24 (1)	36.9 ± 6	F = 1.759	0.174	56.9 ± 10.2	F = 6.281	0.002 *	8.5 ± 5.1	F = 1.686	0.187
25–34 (2)	38.4 ± 7.1			60.7 ± 9.1	^2 > 1^ **	0.04	8.4 ± 5.6		
35+ (1)	38 ± 1.8			56.7 ± 7.7			4.3 ± 1.9		
**Educational status**									
Primary education (1)	36.8 ± 6.6	F = 21.01	<0.001 *	56.4 ± 7.7	F = 32.3	<0.001 *	10.1 ± 5.5	F = 15.1	< 0.001 *
High school (1)	35.8 ± 6.3	^2 > 1^ **	0.114	56 ± 9.8	^2 > 1^ **	0.121	9.8 ± 5.6	^2 > 1^ **	0.108
University (2)	40.6 ± 6.4			64.1 ± 7.7			6.3 ± 4.5		
**Spouse’s educational status**									
Primary education (1)	37 ± 6.6	F = 16.2	<0.001 *	57.2 ± 9.3	F = 36.2	<0.001 *	10.6 ± 5	F = 15.3	<0.001 *
High school (1)	36.1 ± 6.2	^2 > 1^ **	0.089	56.9 ± 9.3	^2 > 1^ **	0.139	8.9 ± 5.6	^2 > 1^ **	0.085
University (2)	40.5 ± 6.6			64.7 ± 7.5			6.5 ± 4.9		
**Place of residence**									
Urban	39.1 ± 7	t: 3.841	<0.001 *	62 ± 8.9	t: 5.969	<0.001 *	7.1 ± 5.3	t: −4.845	<0.001 *
Rural	36.4 ± 6		0.04	56.1 ± 9.4		0.10	10 ± 5.3		0.07
**Family type**									
Nuclear	38.4 ± 6.4	t: 2.592	<0.001 *	60.1 ± 9.1	t: 2.469	0.014 *	7.8 ± 5.2	t: −3.838	<0.001 *
Extended	36.1 ± 7.7		0.02	56.9 ± 10.7		0.018	10.6 ± 5.9		0.04
**Employment status**									
Working	38.2 ± 6.7	t: 1.892	0.059	60 ± 9.4	t: 1.885	0.054	8.2 ± 5.5	t: −1.09	0.276
Not working	36.1 ± 6.7			58.4 ± 9.8			9.2 ± 4.8		
**Family income status**									
Less (1)	36.8 ± 6.6	F = 12.0	<0.001 *	56.3 ± 9.6	F = 19.2	<0.001 *	10.3 ± 5.6	F = 11.6	<0.001 *
Equals (1)	37.9 ± 6.2		0.07	58.9 ± 9.3		0.10	7.1 ± 5.3		0.07
More than (2)	40.7 ± 7.2			63.8 ± 7.9			6.5 ± 4.8		
**Duration of marriage**									
1–5 years	37.8 ± 6.8	F = 1.458	0.234	59.2 ± 9.6	F = 1.024	0.36	8.6 ± 5.5	F = 2.416	0.091
5–10 years	38.1 ± 6.7			60 ± 9.9			6.6 ± 5.6		
10+	41.2 ± 5.1			63.2 ± 7.9			6.7 ± 1.7		
**Planned pregnancy status**									
Yes	38.3 ± 6.8	t: 2.012	0.045 *	60.7 ± 9.2	t: 5.158	<0.001 *	8.2 ± 5.6	t: −1.114	0.266
No	36.4 ± 6.4		0.012	54 ± 9		0.07	9.1 ± 4.6		
**Complications in the spouse’s pregnancy**									
Yes	36.1 ± 5.6	t: −1.963	0.05 *	56.3 ± 8.5	t: −2.453	0.015 *	7.3 ± 3.6	t: −1.488	0.138
No	38.2 ± 6.9		0.011	60 ± 9.6		0.02	8.5 ± 5.7		
**Infant’s sex**									
Female	38 ± 6.9	t: 0.215	0.83	59.1 ± 9.7	t: −0.694	0.488	8 ± 5.8	t: −1.434	0.152
Male	37.9 ± 6.6			59.8 ± 9.4			8.8 ± 5.1		
**Spouse’s attendance at antenatal visits**									
Yes	38.6 ± 6.5	t: 4.946	<0.001 *	60.6 ± 9.2	t: 5.71	<0.001 *	7.9 ± 5.3	t: −4.085	<0.001 *
No	33.5 ± 6.3		0.07	52.3 ± 8.4		0.09	11.3 ± 5.5		0.06
**History of depression**									
Yes	35.1 ± 6.7	t: −2.829	0.005 *	58.2 ± 10.1	t: −0.847	0.398	10.5 ± 5.6	t: 2.55	0.011 *
No	38.3 ± 6.7		0.02	59.6 ± 9.5			8.1 ± 5.4		0.02

$\bar{x}$ ± SD: mean ± standard deviation; F: ANOVA test; KW: Kruskal–Wallis H test; t: independent samples *t*-test; η^2^: Eta-squared effect size; ** post hoc comparisons between categories (Bonferroni-adjusted); * *p* < 0.05, MLS: Marital Life Scale, PAAQ: Paternal-Antenatal Attachment Questionnaire, EPDS: Edinburgh Postpartum Depression Scale. For the F and KW tests, df1: 2, df2: 333; for the *t*-test, df: 334 (Levene’s test > 0.05).

**Table 4:** Multiple linear regression analysis results of independent variables affecting EPDS scores.

	β	SE	LL	UP	t	*p*
(Constant)					12.612	<0.001
MLS	−0.273	0.061	−0.393	−0.153	−4.470	<0.001 *
Educational status (0 = Primary education, High school; 1 = University)	−0.166	0.081	−0.326	−0.006	−2.037	0.042 *
PAAQ	−0.136	0.063	−0.260	−0.012	−2.146	0.033 *
Family type (0 = Extended, 1 = Nuclear)	−0.124	0.049	−0.220	−0.028	−2.538	0.012 *
History of depression (0 = No, 1 = Yes)	0.105	0.048	0.011	0.199	2.182	0.030 *
Spouse’s educational status	0.092	0.080	−0.064	0.248	1.157	0.248
Place of residence	−0.083	0.058	−0.197	0.031	−1.427	0.155
Family income status	−0.039	0.052	−0.142	0.064	−0.744	0.457
Spouse’s attendance at antenatal visits	−0.032	0.051	−0.132	0.068	−0.625	0.533

Model summary = F = 14.4, df1(2): 9(326), *p*: 0.000 *, *R*: 0.533, *R*^2^: 0.284, *R^a^*: 0.264, AIC = 2000, BIC = 2042, VIF = 1.06–3.14, Tolerance = 0.318–0.945, Durbin-Watson = 2.09 (*p* = 0.386). β: Standardized beta coefficient; SE: Standardized standard error; LL–UL: Lower and upper limits of the 95% confidence interval (CI) for standardized β; t: Independent samples *t*-test; F: ANOVA; df: Degrees of freedom; R^a^: Adjusted R^2^; * *p* < 0.05. MLS: Marital Life Scale, PAAQ: Paternal-Antenatal Attachment Questionnaire.

## Data Availability

The de-identified participant data and the data dictionary supporting the reported results are available from the corresponding author upon reasonable request. Data are not publicly archived because the dataset includes information that could permit re-identification of participants in this single-centre cohort. Data sharing is subject to approval by the institutional ethics committee and to completion of a data-use agreement.
